# Male fire ant neurotransmitter precursors trigger reproductive development in females after mating

**DOI:** 10.1038/s42003-021-02921-5

**Published:** 2021-12-15

**Authors:** Robert K. Vander Meer, Satya P. Chinta, Tappey H. Jones, Erin E. O’Reilly, Rachelle M. M. Adams

**Affiliations:** 1 USDA/ARS, CMAVE, 1600 SW 23rd Drive, Gainesville, FL 32608 USA; 2 Foresight Science and Technology, Hopkinton, MA 01748 USA; 3 Virginia Military Institute, Department of Chemistry, Lexington, VA 24450 USA; 4 Ohio State University, Department of Evolution, Ecology and Organismal Biology, Columbus, OH 43212 USA; 5 National Museum of Natural History, Department of Entomology, Washington, DC 20560 USA

**Keywords:** Entomology, Enzymes, Chemical ecology

## Abstract

Social insect queens have evolved mechanisms to prevent competition from their sexual daughters. For *Solenopsis invicta*, the fire ant, queens have evolved a primer pheromone that retards reproductive development in their winged reproductive daughters. If these daughters are removed from the influence of the queen, it takes about a week to start reproductive development; however, it starts almost immediately after mating. This dichotomy has been unsuccessfully investigated for several decades. Here we show that male fire ants produce tyramides, derivatives of the biogenic amine tyramine, in their reproductive system. Males transfer tyramides to winged females during mating, where the now newly mated queens enzymatically convert tyramides to tyramine. Tyramine floods the hemolymph, rapidly activating physiological processes associated with reproductive development. Tyramides have been found only in the large Myrmicinae ant sub-family (6,800 species), We suggest that the complex inhibition/disinhibition of reproductive development described here will be applicable to other members of this ant sub-family.

## Abstract (web summary)

Vander Meer et al. investigate the mechanisms of male involvement in reproductive development in fire ant newly mated queens. The authors find that males produce tyramide compounds that, when transferred to females during mating, are converted to bioactive molecules that trigger wing loss, ovary development, and queen pheromone production.

## Introduction

In the order Hymenoptera, bee, and wasp species may be solitary or social, whereas all ant species are social. At the highest social level, eusociality, there is reproductive division of labor where the queen(s) monopolizes reproduction and daughters work cooperatively to care for colony members: queen, workers, and winged reproductive females and males (alates) over several generations. Ants occupy virtually every terrestrial ecological niche in the world and have evolved multiple strategies to achieve this broad success. In concert with the evolution of eusociality, ants have developed a variety of exocrine glands and associated semiochemicals that support sophisticated levels of coordination among colony members. For example, releaser pheromones elicit a direct behavioral response, e.g., alarm pheromones, while primer pheromones^1^ can take weeks to manifest a measurable physiological effect, e.g., queen inhibition of female sexual production^2^. Many releaser pheromones have been isolated and identified from social insects^1^; however, primer pheromones have been less tractable. One exception being the highly investigated honeybee^2^, due to their importance as a pollinator of food crops. The second most studied social insect is the invasive fire ant, *Solenopsis invicta* (Buren), because of its global negative impact on a wide range of economic sectors^3^. Bioassays demonstrated the existence of fire ant queen produced primer pheromones, e.g., inhibition of winged female production^4^, and regulation of nestmate recognition in workers^5^; however, no primer pheromones have been identified.

The driving force for this article started with the discovery that fire ant queens release a dealation (wing loss) inhibitory primer pheromone that prevents their winged female daughters from becoming reproductively active while in their mother’s colony^6^. Disinhibited winged females undergo the same physiological changes that occur after mating: dealation, wing muscle histolysis, ovariole development, and queen pheromone production^7,8^. If these events occurred within the mother colony, the queen’s daughters would compete with her for resources, produce male-destined un-inseminated eggs, and produce queen pheromones that would act to diminish the egg-laying rate of their mother^9^. However, when environmental conditions are right, males and primer pheromone inhibited winged females participate in a coordinated population-wide mating flight^10^. This action removes the winged female/newly mated queen (NMQ) from the influence of her mother’s primer pheromone(s) and within minutes of mating and landing the reproductive physiological processes outlined above commence, and after landing she digs a nuptial chamber to start a new colony.

If winged females are removed from the influence of their queen it takes 1.5–6 d^11^ for wing loss to occur, which is congruent with primer pheromone activity that may take days or weeks for the effects to disappear. It was proposed that the dealation inhibitory primer pheromone keeps juvenile hormone levels low in winged females, while in their mother colony^12–14^. Indeed, topical application of juvenile hormone to isolated winged females decreased the time to dealation to about 9 h^15^. In contrast, dealation occurs conservatively within 30 min after winged females mate^10^ and land on the ground, 18 times faster than with juvenile hormone treatment (see Supplementary Fig. 1a). This time difference suggests that dealation caused by primer pheromone disinhibition (1.5–6 d), and dealation after a mating flight (<30 min) are accomplished through different mechanisms^11,15^.

Fire ant mating flights occur after a recent rain, low wind, and temperatures between about 24–33 °C^10^. Flights start when excited males, winged females, and workers open the mound. This is followed by male and/or winged female flight initiation. Ultimately, winged females mate in the air with a single male and become newly mated queens (NMQs)^16,17^ (see Supplementary Fig. 1b). NMQs land and usually quickly break off their wings and search for a suitable place to start a new colony. Winged females collected at the beginning of mound activation (prior to flight), or after laboratory-induced flight activity, did not remove their wings faster than alates removed from the influence of their queen^14^, leaving the mating process as the trigger to rapid wing loss and reproductive development. Mating cannot be induced in the laboratory; however, changes in the chemistry of winged females and NMQs, before and directly after mating (<30 min) can be informative^18^.

While the queen dealation inhibitory primer pheromone helps the queen maintain reproductive dominance, rapid release from reproductive inhibition after mating is necessary for her daughters. The NMQ digs a nuptial chamber and seals off the opening. She then relies on her fat body, crop contents, and wing muscle histolysis products as nutritional resources to rear her first brood. This is a fine-tuned process, mistakes can be fatal, including waiting up to 6 days to start reproductive development. We hypothesized that a mechanism evolved to quickly start reproductive physiological changes in NMQs.

Tyramides, are a class of compounds reported from male ant gasters (the gaster starts with abdominal segment 3, abdominal segments 1 and 2 form the petiole in ants)^19,20^. Here we identify tyramides for the first time from *S. invicta* males and elucidate the extraordinary mechanism that has likely co-evolved due to male and female interactions in this species, to neutralize the effect of the queen on her sexual daughters. The sequence of events begins with reproductively inhibited winged females and males participating in a mating flight. During mating, male biosynthesized tyramides are transferred to winged females. The winged females produce an enzyme in their reproductive system that hydrolyzes the transferred tyramides to tyramine, a biogenic amine neuromodulator^21^. The tyramine produced, floods the hemolymph of NMQs, overcoming the effects of the queen’s primer pheromone and accelerating reproductive development.

## Results

### Unique male tyramide chemistry

**Fig. 1:**
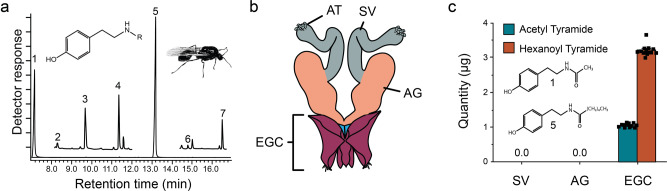
Unique male tyramide chemistry. **a** GC-MS trace of fire ant male produced tyramides (**1** through **7**), minor tyramide components (2, 3, 4, 6, 7) are shown on a reduced scale above the baseline for visualization. A general tyramide structure and a winged male are shown. **b** The unusual tyramide chemistry is specific to the male reproductive system (AT Atrophied Testes; SV Seminal Vesicles; AG Accessory Glands; EGC External Genitalia Complex. The EB, Endophallic Bladder resides within the EGC). **c** Further, analyses of the three distinct male reproductive system parts for tyramides showed they are found exclusively in the EGC (*n* = 13) Structures for the two major tyramide components (**1** & **5**) are shown. Bars represent the mean ± SEM and squares represent individual males. See Table 1 for the percent composition and quantity of the seven identified tyramides.

Initially, we qualitatively identified seven homologous n-alkyl-tyramides (1–7, Fig. 1a and Table 1, acetyl- to octanoyl-tyramide) from crushed male gasters and then the tyramides were identified from an extract of male reproductive systems (Fig. 1b)^22^. The two major tyramides (acetyl- and hexanoyl-) comprise >96% of the identified tyramides and are the focal compounds for this study (Table 1). Sperm produced in the testes is stored in male seminal vesicles. The testes atrophy after sperm transfer (Fig. 1b)^23^. Other discrete male reproductive system parts are accessory glands, and the endophallic bladder, which is integrated within the external genitalia complex (Fig. 1b). Analyses of the seminal vesicles, accessary glands, and the external genitalia complex for tyramides showed that acetyl- and hexanoyl- tyramides are found exclusively in the external genitalia complex (Fig. 1c): 1.05 ± 0.01 µg (Brown−Forsythe ANOVA test *F*_2,12_ = 5504, *p* < 0.0001, *n* = 13) and 3.2 ± 0.04 µg (Kruskal Wallis H_2_ = 36.02, *p* < 0.0001), respectively, *n* = 13). Acetyl- and hexanoyl-tyramide structures are shown in Fig. 1c.

**Table 1:** Tyramide identification and quantity.

Tyramide	RT^a^	*R* =	Percent ± SE	µg/male ± SE
1	7.162	Acetyl^b^	23.86 ± 0.35	1.05 ± 0.01
2	8.325	Propanoyl^b^	0.23 ± 0.01	<0.01
3	9.718	Butanoyl^c^	0.32	0.02^c^
4	11.378	Pentanoyl^b^	2.01 ± 0.04	0.09 ± 0.001
5	13.170	Hexanoyl^b^	72.73 ± 0.37	3.2 ± 0.04
6	14.777	Heptanoyl^b^	0.23 ± 0.01	<0.01
7	16.446	Octanoyl^b^	0.45 ± 0.01	0.02 ± 0.0004

Qualitative GC retention time (RT) data are shown for the seven identified tyramides. Quantitation (μg/male) for tyramide components 1, 2, 4, 5, 6, and 7 from GC-MS analyses of external genitalia complex extracts (*n* = 13 independent external genitalia complex samples) were obtained using n-butanoyl tyramide as a relative internal standard. Peak assignments were based on fragmentation pattern, retention time, and standards (THJ^20^). The percent of n-butanoyl tyramide of the 7 tyramides was determined from a single sample of five pooled male reproductive systems using the octanoyl tyramide (synthesized by THJ^20^) as the External Standard (ES). Percent composition was based on the values in the µg/male column. See Fig. 1a for the GC-MS chromatogram profile.

^a^Retention time.

^b^From Internal Standard method, *n* = 13.

^c^From External Standard method.

### Males control tyramide release

**Fig. 2:**
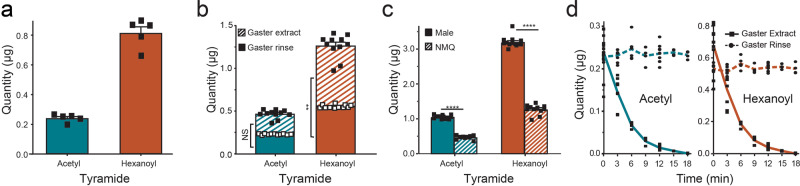
Tyramide transfer from male to winged females during mating and tyramide loss. **a** Acetyl- and hexanoyl-tyramides were recovered from Newly Mated Queen (NMQ) gaster tip rinses and quantified (*N* = 5). **b** In a separate experiment, tyramide quantities were determined from NMQ gaster tip rinses and gaster extracts from the same NMQs (paired T-test, Wilcoxon match-pairs signed ranks test, *n* = 11). **c** The amount of tyramides available from the male (*n* = 13) are compared with the total amount recovered from NMQs (*n* = 11, *t* = 0) using a T-test (acetyl-) and Mann−Whitney test (hexanoyl-) to account for non-gaussian data. **d** The loss of tyramides transferred to winged females during mating from gaster rinses and gaster extracts was measured over time, from 0 (time at collection) to 18 min at 3 min intervals. Replicates for the 7 time periods were: 11, 8, 6, 4, 10, 4, 4, respectively. All replicate values represent biologically independent samples. Tyramides from gaster rinses did not change with time; however, tyramides from gaster extracts were undetectable or in trace amounts by 18 min. In all plots, acetyl tyramide values are represented by teal bar/line, hexanoyl tyramide by orange bar/line, points represent individual NMQs or Males. **p < 0.01; ****p < 0.0001. Error bars = SEM.

The flow of material from the male reproductive system may be simultaneous, where seminal vesicles, accessory glands, and external genitalia complex contents flow together into the female reproductive system during mating (see Supplementary Fig. 2aI and aII, black arrow), or males control the release of tyramides from the endophallic bladder/external genitalia complex. Detailed diagrams of the male reproductive system are informative (Supplementary Fig. 3a–d). The pair of seminal vesicles and accessory glands merge into the ejaculatory duct at the anterior corners of a sclerotized wedge; however, the endophallic bladder contents enter the ejaculatory duct near the posterior apex of the sclerotized wedge (Supplementary Fig. 3c, d)^22^. Therefore, the release of the endophallic bladder contents can be independent of the release of seminal vesicle and accessory gland contents. Further to the question of whether males control the release of tyramides, we hypothesized that if the seminal vesicles, AC, and endophallic bladder/external genitalia complex contents are simultaneously released into winged females, then the newly mated queen (NMQ) spermatheca (sperm storage/dispensing organ, see Supplementary Fig. 2aI) should contain detectable amounts of tyramides. Spermatheca were dissected from NMQs (*n* = 5), and each analyzed for the presence of tyramides by GC-MS. No tyramides were detected in these samples, in support of the male release of tyramides after the release of seminal vesicle and accessary gland contents. Release of the endothallic bladder contents likely further push the seminal vesicles and accessory glands contents toward and into the spermatheca. Furthermore, if tyramides are released at the end of the mating process, we predicted that measurable amounts of tyramides would be found on the tip of NMQ gasters, since the volume of the winged female reproductive system is finite. Analyses for tyramides in methanol rinses of NMQ gaster tips (see Supplementary Fig. 2b) resulted in, 0.24 ± 0.01 µg of acetyl tyramide and 0.81 ± 0.04 μg of hexanoyl tyramide (*N* = 5), in support of endophallic bladder/external genitalia complex tyramide release at the end of the mating process (Fig. 2a). Separately (Fig. 2b), tyramides were quantified from NMQ gaster tip rinses and gaster extracts, both from the same NMQ. The amount of acetyl tyramide found in the gaster extract (0.24 ± 0.015 µg) did not differ significantly from the quantity found in the tip wash (0.23 ± 0.002 µg) (difference of 0.01 ± 0.01 µg, two-tailed paired T-test, *t*_10_ = 0.73, *p* = 0.48, *N* = 11). The amount of hexanoyl tyramide found in gaster extracts (0.71 ± 0.04 µg) was significantly higher than the amount from tip rinses (0.56 ± 0.006 µg) (difference of 0.15 ± 0.04 µg, Wilcoxon match-pairs signed ranks test, *V* = 3, *p* = 0.0049, *N* = 11). All *n*/*N* values represent biologically independent samples.

Comparison of available male tyramides (Fig. 1c) with total tyramides recovered from NMQs (gaster extracts + gaster rinses, Fig. 2c) showed only 45% acetyl- (0.47 ± 0.02 µg) and 40% hexanoyl- (1.26 ± 0.04 µg) tyramide recovery, indicating a significant loss of available male tyramides from NMQs prior to landing and collection (acetyl-: difference of 0.58 ± 0.02 µg, two-tailed T-test, *t*_22_ = 27.67, *p* < 0.0001; and hexanoyl-: difference of 1.93 ± 0.06 µg, Mann−Whitney test, *U* = 0.0, *n*1 = 11, *n*2 = 13, *p* < 0.0001, respectively, NMQs, *n* = 11, males, *n* = 13). The lack of material (tyramide) balance could be due to inefficient transfer of tyramides, male to female, or tyramide degradation in the winged female/NMQ reproductive system, or other, unknown reasons. All *n*/*N* values represent biologically independent samples.

### The fate of tyramides transferred from male to female during mating

Tyramide analyses of dissected winged female reproductive systems showed no detectable tyramides (N = 10, independent samples); therefore, tyramides found in newly mated winged females (NMQs) must be derived from the males with whom they mated. Tyramides from gaster tip washes did not change over time, Fig. 2d (acetyl tyramide, 0.23 ± 0.002 μg at *t* = 0 (*n* = 11) to 0.23 ± 0.004 μg at *t* = 18 min (*n* = 4) and hexanoyl tyramide, 0.56 ± 0.006 μg at *t* = 0 (*n* = 11) to 0.56 ± 0.02 μg at *t* = 18 min (*n* = 4). Neither acetyl- or hexanoyl-tyramide from gaster tip washes showed a slope that was significantly different from zero (*F*_1,45_ = 0.72, *p* = 0.40 and *F*_1,45_ = 0.92, *p* = 0.34 respectively, *N* = 47). Tyramide concentrations in NMQ gaster extracts (from the same NMQs used in the NMQ gaster tip rinses) over time (Fig. 2d), showed exponential tyramide loss. Acetyl tyramide, from 0.24 ± 0.02 μg at *t* = 0 (*n* = 11) to 0.0005 ± 0.0005 μg at *t* = 18 min (*n* = 4), *y* = 0.24e^−0.205*x*^, *R*^2^ = 0.91, and hexanoyl tyramide, from 0.71 ± 0.04 μg at *t* = 0 (*n* = 11) to 0.002 ± 0.002 μg at *t* = 18 min (*n* = 4), *y* = 0.72e^−0.207*x*^, *R*^2^ = 0.92. Log(*x* + 1) transformed data from the gaster extract showed a significant linear relationship for acetyl- and hexanoyl-tyramide (*F*_1,45_ = 195.0, *p* < 0.0001, *R*^2^ = 0.81; and *F*_1,45_ = 237.6, *p* < 0.0001, *R*^2^ = 0.84 *N* = 47), respectively. Tyramides were undetectable in gaster extracts by 18 min after NMQs were collected, except for a single NMQ with trace amounts of tyramides (acetyl 0.002 μg and hexanoyl 0.007 μg). All *n*/*N* values represent biologically independent samples. A tyramide degradation mechanism likely exists in NMQ gasters that is not on the gaster surface.

### Start of tyramide degradation

The rapid loss of tyramides (Fig. 2d), and the large discrepancy between the amount of tyramides available from males (Fig. 1c) and tyramide amounts found in newly mated queens (NMQs) directly after landing (Fig. 2c), suggests that tyramide degradation and reproductive development likely commence directly after mating; however, NMQ wing loss cannot occur until the NMQ returns to the ground after mating. Support for this comes from laboratory research where winged females, isolated from their queen, commenced reproductive development, but 27% of these did not lose their wings^6^.

**Fig. 3:**
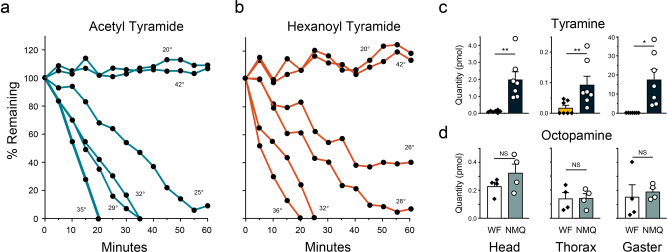
The fate of tyramides transferred from male to female during mating. Extracts of dissected vulva/bursa copulatrix (see Supplementary Fig. 2-I red oval) from winged females (WF) > 15 mg) were incubated with synthetic tyramides **a** acetyl- and **b** hexanoyl-, at various temperatures and the percent loss of tyramides determined over time. **c** Shows the quantity of tyramine in winged female and NMQ parts (head, thorax, and gaster) compared with Welch’s t-test and Mann−Whitney test, *n* = 7; note differing scales on the *y*-axes. **d** Shows the quantity of another biogenic amine, octopamine, from four of the same winged females and NMQ samples used for tyramine analyses (compared with t-test, *n* = 4). Bars represent mean + SEM, circles represent individual NMQs, diamonds represent individual winged females (WF)s, *p < 0.05; **p < 0.01, NS not significant.

A probing experiment that challenged an aqueous extract of the winged female vulva/bursa copulatrix (see Supplementary Fig. 2a–I, female genitalia, red oval) with synthetic tyramides showed rapid tyramide degradation, adding support for tyramide hydrolase activity in the winged female vulva/bursa copulatrix. We then systematically followed the loss of synthetic acetyl- and hexanoyl- tyramide (Fig. 3a, b, respectively), when incubated at various temperatures in an aqueous extract of the vulva/bursa copulatrix from winged females. Enzymatic loss of acetyl tyramide was quickest at about 35 °C; however, no tyramide loss was observed when incubation was <22 °C or >40 °C. For hexanoyl tyramide, degradation occurred fastest at 36 °C, but no loss occurred if the incubation temperature was <25 °C or ≥40 °C. These distinct temperature ranges for tyramide hydrolysis bracket the temperature range reported for mating flight initiation^10^, suggesting an enzyme system that likely co-evolved in winged females along with the male biosynthesis of tyramides.

The uniqueness of the proposed “tyramide hydrolase” was supported in experiments that incubated acetyl- (32 and 35 °C) and hexanoyl-tyramide (32 and 36 °C) at optimal hydrolysis temperatures (Fig. 3a, b), with an extract of winged female digestive tract. Tyramides were unaffected by digestive enzymes (see Supplementary Fig. 4). Similarly, incubation of acetyl- (32 and 35 °C) and hexanoyl-tyramide (32 and 36 °C) in water did not result in tyramide hydrolysis. Tyramides are stable compounds (see Supplementary Fig. 4).

Logically, tyramide hydrolysis would produce the biogenic amine, tyramine. Indeed, qualitative mass spectral analysis (Advion™ System) of the product from the variable temperature hydrolysis experiments (Fig. 3a, b) verified that tyramine was produced. These data support the presence of a unique enzyme system in winged females. We propose that the role of this system is to hydrolyze tyramides transferred from males to winged females during mating, to tyramine—resulting in accelerated NMQ reproductive development.

Tyramine and another biogenic amine, octopamine^21^ (Fig. 3c, d, respectively), were identified and quantified from the same head, thorax, and gaster samples of winged females and NMQs. Newly mated queens (*n* = 7) had significantly higher amounts of tyramine than winged females (*n* = 7) for the head, thorax, and gaster: winged female-heads, 0.097 ± 0.02 pmol versus NMQ-heads, 1.97 ± 0.46 pmol (difference of 1.83 ± 0.46 pmol, Welch’s t-test: *t*_6.03_ = 4.095, *p* = 0.006); winged female-thorax, 0.02 ± 0.008 pmol compared to NMQ-thorax, 0.09 ± 0.03 pmol (difference of 0.073 ± 0.03 pmol, Mann−Whitney test *U* = 4, *n*_1_ = 7, *n*_2_ = 7, *p* = 0.006); and winged female-gaster, 0.02 ± 0.005 pmol versus NMQ-gaster, 15.93 ± 4.64 pmol (difference of 15.91 ± 4.64 pmol, Welch’s t-test: *t*_6_ = 3.43, *p* = 0.014). Figure 3d**:** Quantitation of octopamine from the same winged female (*n* = 4) and NMQ (*n* = 4) samples used for tyramine analyses showed no significant differences: winged female-head, 0.23 ± 0.03 pmol versus NMQ-head, 0.32 ± 0.06 pmol (0.01 ± 0.07 pmol, t-test: *t*_6_ = 1.35, *p* = 0.23); winged female-thorax, 0.14 ± 0.04 pmol versus NMQ-thorax, 0.14 ± 0.03 pmol (0.002 ± 0.06 pmol, t-test: *t*_6_ = 0.045, *p* = 0.97); and winged female-gaster, 0.16 ± 0.09 pmol versus NMQ-gaster, 0.19 ± 0.03 pmol (0.04 ± 0.09 pmol, t-test: *t*_6_ = 0.40, *p* = 0.70). All *n*/*N* values represent biologically independent samples. Tyramine is the biosynthetic precursor to octopamine^21^ and even though tyramine levels are greatly elevated in NMQ abdomen samples, octopamine levels remain unaffected. The special biosynthesis of tyramides by males coupled with a specific tyramide hydrolase in winged females and high levels of tyramine in NMQ hemolymph, support a specific role for tyramine in NMQs. We propose that tyramine overrides the effects of the queen’s dealation inhibitory primer pheromone.

## Effects of tyramine injection on winged females

Our original goal was to answer the question: how are NMQs able to break out of the influence of their mother queen so much faster than isolated winged females? The above results allow us to further hypothesize that when tyramine floods the NMQ gaster it activates tyramine receptors that are linked to wing loss, ovariole development, and pheromone production. The following experiments test this hypothesis.

### Wing loss (dealation)

**Fig. 4:**
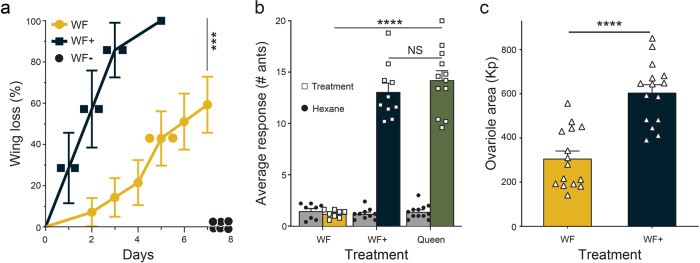
Phenotypic effects of tyramine injection into winged females. **a** Tyramine (aqueous solution) injected into winged females (WF+, *n* = 7) leads to quicker dealation than for winged females injected with water (WF, *n* = 14). Eight winged females injected with water lost their wings by day 7 (yellow circles); however, six winged females injected with water did not dealate within the 7-day experiment (WF−, black circles on *x-*axis). Data analyzed by Log-rank, Mantel−Cox test). **b** Bar on left of each of the three pairs of bars is the worker response to the hexane control. Venom sac extracts from tyramine injected winged females (WF+, *n* = 10) resulted in 1) a significantly higher number of responding worker ants than for venom sac extracts from saline injected winged females (n = 8), and 2) the number of responding worker fire ants was not different from worker response to mature queen poison sac extract (*n* = 12)^24^. Comparisons made with Brown−Forsythe ANOVA (with Dunnett’s T3 MCT post hoc). **c** Tyramine injected into winged females (*n* = 15) led to significantly larger ovarioles compared to ovarioles from water-injected winged females (*n* = 15, comparison made with t-test). All *n* values represent biologically independent samples. Bars represent mean ± SEM, ***p < 0.001; ****p < 0.0001, NS not significant.

An aqueous solution of tyramine injected into winged females resulted in significantly faster dealation and a higher percentage of dealation than for winged females injected with water (Fig. 4a) (Kaplan–Meier survival/mortality curves, Log-rank, Mantel−Cox test χ^2^ = 12.83, *p* = 0.00031 (*n* = 7 treatment, *n* = 14 water control). Eight of the 14 water control winged females lost their wings by the end of the 7-day experiment; however, 6 water controls did not lose their wings and are shown as black circles on the *x*-axis in Fig. 4a. All *n*/*N* values represent biologically independent samples.

### Queen pheromone production

Worker aggregation responses to venom sac hexane extracts derived from (a) tyramine injected winged females (WF+), (b) saline injected winged females (WF), and (c) positive control—polygyne queen poison sac hexane extracts (queen poison sacs contain worker attractants^24^) were significantly different (Brown-Forsythe ANOVA test F_5,23.47_ = 142.8, *p* < 0.0001, winged females (WF, *n* = 8), winged females+ (*n* = 10), queens (*n* = 12), Fig. 4b. Significantly more worker ants responded to treatment (a) vs. control (b): 13.08 ± 0.85 vs 1.20 ± 0.13 workers responding, Dunnett’s T3 multiple comparisons test: mean difference −11.88 ± 0.85, *t* = 13.86, df = 9.4, *p* < 0.0001, *n* = 10 WF+, 8 WF. Worker response to control (b) was significantly less than the response to positive control (c): (1.20 ± 0.13 vs. 14.23 ± 0.90 workers responding, Dunnett’s T3 MCT: mean difference −13.03 ± 0.91, *t* = 14.27, df = 11.46, *p* < 0.0001, Winged females (WF, *n* = 8) and queens (*n* = 12). Worker response to treatment (a) was not different from the positive control (c): (13.08 ± 0.85 vs. 14.23 ± 0.90 workers responding, Dunnett’s T3 MCT: mean difference −1.15 ± 1.23, *t* = 0.93, df = 19.98, *p* = 0.73, winged female (WF+, *n* = 10) and queens (*n* = 12). Welch’s t-test was used to correct for the unequal variances when WF+ and queen results were compared to their respective hexane controls: In both situations the worker response to queen and to winged female+ (WF+) were significantly higher than their hexane controls. Queen vs. hexane control, difference of 12.83 ± 0.92, Welch’s t-test, *t*_12.01_ = 13.88, *p* < 0.0001, *n* = 12. Winged female+ (WF+) vs. hexane control, difference of 11.88 ± 0.86, Welch’s t-test, *t*_9.7_ = 13.75, *p* < 0.0001, *n* = 10. Winged female treatments (saline) vs. their hexane controls were not significantly different, (0.28 ± 0.30, two tailed t-test, *t*_10.24_ = 0.93, *p* = 0.37, *n* = 8). All *n*/*N* values represent biologically independent samples.

### Ovariole development

Ovariole development is another physiological change that occurs after mating. Tyramine injected into low-weight winged females induced significantly greater ovariole development, Fig. 4c (60.5 ± 3.6 Kp, *n* = 15), than saline injected winged females (30.6 ± 3.4 Kp, *n* = 15), −29.9 ± 5 Kp, t-test, *t*_28_ = 5.98, *p* < 0.0001, *N* = 30. All *n*/*N* values represent biologically independent samples.

When the level of tyramine is elevated in winged females through mating and tyramide conversion or through tyramine injection, physiological events associated with reproductive development are activated, e.g., wing loss, queen pheromone production, and ovariole development.

## Discussion

Eusociality in mammals is fully expressed only by naked mole rats, where all members of the colony are the queen’s progeny. She releases hormones that suppress sexual development in her daughters and all but a few male offspring^25^. Colony members forage for resources and care for the young produced by their queen for the lifespan of the queen/colony (~30 yrs.). Interestingly, essential maternal care of the queen’s offspring by sexually suppressed females is promoted by female consumption of queen feces, which are rich estradiol that induces maternal care behaviors, but not sexual development^26^.

Like the naked mole rat, eusocial hymenopteran queens (some wasps and bees and all ants) need to control reproductive competition in the colony. For the honeybee, workers emerge as adults with immature ovaries. The queen releases primer pheromones that suppress worker reproductive development. If the queen dies, worker ovaries mature, and they produce males (drones) from unfertilized eggs. In large colonies workers may evade the effects of primer pheromone and lay eggs, but sisters attack them. In addition, workers can distinguish eggs laid by workers or the queen and kill worker laid eggs, this action has been designated as worker policing^2^. Worker policing and queen inhibition of worker reproduction are similarly found in the eusocial wasp, *Vespula vulgaris*^27^. Eggs laid by uninhibited worker wasps are recognized as different and are killed by workers. The “policing” mechanism reduces conflict within the colony and helps maintain the queen’s reproductive monopoly. Policing even occurs in *Cerapachys biroi*, an ant species that reproduces clonally, and workers are genetically identical^28^. Here, worker reproduction is inhibited by larval pheromone cues that also alter worker cuticular chemistry. Non-inhibited workers could lay eggs, but they are recognized as different and are executed (policed) by inhibited workers, maintaining reproductive harmony in the colony.

The general theme in these examples is that reproductive competition is detrimental to the colony unit and is selected against in eusocial insects. The fire ant presents similar yet different challenges. The research presented here impacts at least four interrelated areas: (1) the queen’s need to suppress reproductive competition; (2) the NMQ’s need to quickly start reproductive development; (3) the male biosynthesis/transfer of tyramides to winged females; and (4) the ability of NMQs to hydrolyze tyramides to the biogenic amine, tyramine^21^—an instant antidote to the queen’s dealation inhibitory primer pheromone.

Fire ants have derived eusocial traits where workers are sterile, devoid of ovaries; therefore, worker “policing” is not necessary. Workers provide resources to colony members, care for the queen, and brood, defend the colony against enemies. However, there is a different problem, mating flight initiation is weather conditional; therefore, winged females could be stuck in the colony for several weeks to months. Activation of their ovaries would be in direct conflict with the colony queen. Like queen inhibition of worker ovary development in previous examples, the fire ant queen inhibits reproductive development in winged females. For the winged females, reproductive inhibition has two positive effects: (A) prevents reproductive competition with the queen and (B) keeps them in a mating flight-ready state.

Inhibition of worker ovary development in the bee, wasp, and ant examples is the physiological state these workers will likely be in their entire life. This is due to the short lifespan of workers compared to their queens. However, the reproductively inhibited fire ant winged females represent future fire ant generations and to be successful they need to quickly deactivate the queen’s inhibitory effects after mating! For NMQs the stakes are high,—they have finite energy reserves, just enough to rear their first brood to adults, and delays in first worker production could be fatal. NMQs show no signs of reproductive inhibition, they quickly start the physiological changes needed for colony foundation, as they descend from their mating flight.

We sought to determine how NMQs are rapidly released from the influence of their mother queen. The discovery of tyramides in male ants was the catalyst to a series of unexpected discoveries: (a) tyramides were found specifically in the male endophallic bladder, not previously reported to have a function; (b) the males control when tyramides are released, at the end of the mating process; (c) winged females produce a tyramide hydrolase in their reproductive system that rapidly converts tyramides to the biogenic amine neuromodulator tyramine^21^, after mating; (d) digestive enzymes have no effect on the tyramides; (e) the tyramide hydrolase is active only at the temperature range reported for fire ant mating flights; (f) tyramine levels are significantly elevated in newly mated queens; (g) injection of tyramine into winged females results in rapid onset of phenotypic events associated with reproductive development. Male-derived tyramides and female production of a temperature-specific enzyme that converts tyramides to tyramine, represents a remarkable evolved system that quickly overrides the effects of the queen pheromone that suppresses winged female reproduction.

Related to our proposed tyramine activation of tyramine G-protein-Coupled receptors, is the mechanism evolved to deactivate the biogenic amine/GPCR complex^29^. Acetylation of the bound biogenic amine releases it from the GPCR and stops the biosynthetic machinery! Acetylated tyramine is the same as acetyl tyramide (compound 1). This deactivation mechanism appears to be general to biogenic amines, since besides acetyl tyramide, acetylated octopamine, dopamine, and serotonin, were also isolated from brain extracts of *Formica japonica* queens^28^. There is precedence in ants for the biosynthesis of tyramides^30^.

There are currently over 16,000 described ant species/subspecies in 17 extant sub-families^31^. The Myrmicinae are the most species rich (~6,830)^31^ and have evolved the ability to biosynthesize a variety of venom alkaloids for defense and food procurement^32^. The Formicinae is the second most specious ant sub-family^33^. They use formic acid for defense and food procurement, and Formicinae males do not produce tyramides^20^. The limited diversity of tyramide structures found in seven genera from two of the largest myrmicine tribes (Solenopsidini with 773 species and Attini with 2,558 species) suggest conservation of at least some compounds^19,20,34^. Therefore, it is likely the work described here has broad applicability, reaching beyond the two tribes, but limited to the Myrmicinae sub-family. All ant queens are faced with the challenge of maintaining reproductive control within the colony, and likewise the sexual females after mating need a mechanism to overcome the influence of the queen and quickly start reproductive processes. We anticipate that many similar tyramide/tyramine examples will be elucidated in the future; however, the grand challenge will be to discover what mechanisms other ant sub-families have evolved to cope with similar queen-daughter conflict.

## Methods

### Statistics and reproducibility

All statistical procedures and graphical representations were carried out using GraphPad Prism, version 9.01 (GraphPad Software Inc., San Diego, CA). Significance is determined as *ρ* < 0.05. All results are reported as mean ± 1 standard error of the mean. All samples were tested for Gaussian distribution using Shapiro−Wilk normality test and an F test was used to compare variances between groups. Kruskal−Wallace or Mann−Whitney tests were used if data was non-normal. Wilcoxon matched-pair signed rank test was used in paired comparisons where one or both variables were non-normally distributed. Welch’s t-test or Brown−Forsythe ANOVA was utilized if unequal variance was present. See specific Methods and Results sections and corresponding Figure caption for details and specific statistical procedure(s). Our detailed descriptions of the dissection procedures are aimed at reproducibility for those that may want to extend our work to other ant species.

### Gas chromatograph-mass spectroscopy analyses

Mass spectral data were obtained from (a) Shimadzu QP-2020 GC-MS (Palo Alto, CA) equipped with an RTZ-5, 30 m × 0.25 mm i.d. column. The instrument was temperature programmed from 60 to 250 °C at 10°/min and held there for 20 min; or (b) Agilent Intuvo 9000 GC system, with a Masshunter Data Acquisition Workstation version 10.0.368 (Santa Clara, CA) equipped with an HP-5 MS ultra-inert non-polar column, 30 m × 0.25 m i.d. column, coupled to a 5977 B mass spectral detector. Injector temperature was set at 250 °C. The oven temperature was programmed at 40 °C for 2 min, then 285 °C at 10 °C/min, followed by a 10 min hold at 285 °C.

### Tyramide identification

Tyramides have a distinctive mass spectral fragmentation pattern, m/z 97, 107, 120 (base peak), and 164. Use of selected ion monitoring (SIM) makes it possible to tentatively identify tyramides in complex mixtures (Fig. 1a, shows results for the m/z 120 fragment). Components 2, 3, 4, 6, and 7 were barely, or not visible in the baseline normalized to the most abundant component (5), therefore, baseline sensitivity was increased in two sections of the chromatogram and placed appropriately above the normalized baseline for the purpose of visualization. Positive tyramide identification was achieved by their mass spectral fragmentation patterns and comparison of gas chromatograph retention times with synthetic tyramide standards. Synthetic standards were prepared by previously published methods^20^ and purified by silica gel chromatography. GC-MS analysis showed them to be greater than 95% pure.

### Tyramide quantitation, external standard

Either the concentration of external standard or the concentration of the extract can be adjusted to be close to each other. All injections were set at 1 µl. A known amount of external standard (ES), n-octanoyl or n-butanoyl tyramide (synthetic)^20^, was also run on the GC-MS using the same operating parameters and at a concentration compatible with the tyramides of interest. Peak areas were manually and/or software calculated. Quantitation was done by comparing the peak area of the ES to the peak areas of tyramide analyses. With sample and external standard volumes equal and 1 μl injections, the peak area from 1 μl of ES represents the total amount of ES. Therefore, (tyramide analyte peak area / ES peak area) × total μg ES = μg of tyramide analyte.

A pooled sample of male reproductive systems (*n* = 5) were crushed with forceps in methanol, vortexed, and centrifuged. The supernatant was transferred to a GC-MS autosampler vial. The seven tyramides were quantified using synthetic n-octanoyl tyramide^20^ as an external standard (1 μg/μL) and GC-MS instrument “a”. The chromatogram, Fig. 1a and the quantitative data for n-butanoyl tyramide (Table 1) were derived from these data since n-butanoyl tyramide was used as a relative internal standard for quantitation of the other six identified tyramides (see below). The external standard method (ES = n-butanoyl tyramide) was used to quantitate tyramides in the NMQ gaster methanol rinse experiment, Fig. 2a using GC-MS Instrument “b”. The relative internal standard method was used for all other tyramide quantitation.

### Tyramide quantitation, internal standard

A known amount of internal standard (IS), n-butanoyl tyramide (synthesized by THJ^20^), was added to samples early in the procedure and in an amount measurable with the expected analytes (n-butanoyl tyramide peak integration was carried out manually to increase accuracy. The peak area of the IS represents the original amount added; therefore, analyte peak area/IS peak area × IS amount = amount of tyramide analyte. The n-butanoyl tyramide is a good IS for quantitation of the tyramides, because it: (a) has an intermediate retention time relative to the other six tyramides; (b) has an analogous MS fragment pattern; (c) shows good detector response/concentration linearity (standard curve: *y* = 1130*x* − 2255; *R*^2^ = 0.9735; *p* = 0.0003). and (d) is a minor tyramide component (<1%).

### Tyramides in males

For all dissections, males were randomly chosen from laboratory-reared monogyne fire ant colonies. *Male gasters*. The male reproductive system occupies most of the gaster volume^22^, therefore, gasters were removed from the rest of the body, macerated in methanol, and vortexed or sonicated for subsequent qualitative analyses for tyramides. Qualitative analysis using GC-MS method “b”, demonstrated that tyramides were present in male gasters.

### Dissections—general

A glass microscope slide (75 mm × 25 mm × 1 mm) (C & A Scientific, Manassas, VA) was placed within a petri dish (8.5 cm dia. × 1 cm tall) that had a 5 mm-thick layer of silicone covering the bottom. All dissections were carried out in a 40 μL droplet of DNA/RNA free water placed on the glass microscope slide. A Leica MZ 8 microscope and two sharpened Dumont Inox #5 (Fine Science Tools, Forester City, CA) dissecting forceps were used throughout.

### Male reproductive system—total

A male was pinned ventrally through the thorax and into the silicone base such that the gaster rested on the glass slide near the droplet of water. Both pairs of forceps were used to simultaneously grasp the second or third sternite and gently pull away the gaster contents from the pinned ant. The entire male reproductive system came out intact, with some sternites, tergites and other tissues and organs still attached. This mixture was placed in the 40 µl droplet of water. All non-reproductive system parts were manually separated and removed, leaving the intact reproductive system. The whole male reproductive system was transferred with forceps to an autosampler vial containing 30 μL of methanol (Merck, SupraSolv for gas Chromatography-Mass Spectroscopy, Billerica, MA), and the tissue crushed with forceps, then 30 μL of methanol was used to rinse the forceps into the autosampler vial. The process was repeated until the number of replicates needed was complete. Qualitative analysis of extracts of total male reproductive systems using GC-MS method “b” demonstrated that tyramides are produced in the male reproductive system.

### Dissection of the male reproductive system parts

The procedure for dissection of the male reproductive system was a starting point for dissection of seminal vesicles and accessory glands, see Fig. 1c. After the male reproductive system was removed and placed in the water droplet, the external genitalia (EG) was grasped with forceps and gently pulled until the reproductive system became elongated, exposing the three major parts. The seminal vesicles and accessory glands can be cleanly dissected from the other parts, however, to minimize potential cross-contamination only a pair of seminal vesicles or accessory glands were taken per male.

#### Seminal vesicle dissection

One seminal vesicle was pinched with both forceps where it joined the accessory gland. The pair of forceps closest to the accessory gland were held stationary, and the second pair of forceps gently pulled away from the first pair to separate it from the rest of the reproductive system. The seminal vesicle was removed from the droplet of water and placed on a clean, dry portion of the glass slide with residual water. The second seminal vesicle was similarly removed and placed with the first. One pair of forceps was used to remove excess water from the two seminal vesicles via capillary action then they were both picked up off the slide with the other pair of forceps. Next, they were crushed by opening and closing the single pair of forceps holding them multiple times until a uniform consistency of tissue and fluid was obtained (about five opening and closings), and then the contents were transferred into 30 μL methanol in a 200 μL insert held in a GC autosampler vial. After transferring the crushed seminal vesicles to the methanol solvent, the tips of the pair of forceps were agitated in the insert methanol solvent to remove material left on the forceps. This dissection to methanol solvent time was <30 s. The seminal vesicle samples included atrophied male testes (see AT Fig. 1b).

#### Accessory gland dissection

The accessory gland was separated from the seminal vesicle by pinching and pulling the seminal vesicle closest to where it connects to the accessory gland. The disconnected seminal vesicle was discarded, the remaining accessory gland was then grasped with two pairs of forceps where tissue enters the external genitalia and was gently pinched and pulled away, and placed on the glass slide as described above. The second accessory gland was removed identically and placed on the glass slide with the first accessory gland. As described above, excess water was removed from both accessory glands, then they were crushed and placed in 30 μL of methanol contained in a 200 μL insert held in a GC autosampler vial, as described for the seminal vesicles.

#### External genitalia dissection

The endophallic bladder^35^, formerly known as the aedeagal bladder^22^ is situated within the sclerotized base of the external genitalia. Thus, we are referring to the endophallic bladder within the external genitalia as the external genitalia complex. The bladder has many points of adhesion to the sclerotized base making its intact dissection difficult. Accordingly, the dissection procedure only involved separating the external genitalia complex from the rest of the ant. After a male was pinned ventrally upwards, one side of the male’s parameres (sclerotized clasping structure) is firmly grasped and pulled away from the pinned ant. This method usually resulted in the external genitalia complex separating from the rest of the male reproductive system. After the external genitalia complex was removed, it was crushed with a pair of forceps and placed in a 200 μL insert containing 30 μL of methanol and held in a GC autosampler vial. Another 30 μL of methanol were used to rinse the forceps into the insert. A total of 13 replicates were obtained for each of the three male reproductive system parts.

### Tyramide analyses of male reproductive system parts

Directly after each of the above dissections the n-butanoyl tyramide relative internal standard (2 μg) was added to the inserts held in GC autosampler vials. Samples were analyzed by GC-MS “b”. See Fig. 1c for acetyl- and hexanoyl-tyramide quantitative results for the three male reproductive system parts, *n* = 13. These same samples were used to generate relative internal standard quantitative results for 6 of the 7 tyramides (see Table 1). Butanoyl-tyramide was excluded, since it was the internal standard; however, quantitation of n-butanoyl tyramide was achieved using the external standard method (see above).

### Winged female reproductive system dissection and tyramide analysis

The reproductive system in a mature female alate (winged females, >15 mg) resides entirely at the distal end of the gaster with the ovaries being closer to the midsection. A winged female was placed on a glass slide (along with a few 40 μL droplets of DI water) and the gaster separated from the rest of the body by pulling in opposite directions on the petiole with two pairs of forceps. During dissection, the gaster needs to be stabilized: (A) If the petiole attached to the gaster is intact, it can be used to stabilize the gaster during dissection, or (B) if the petiole is not intact, the gaster can be stabilized by grasping the first sternite or tergite with a pair of forceps. Once separated, place the gaster in one of the droplets of water. For A or B, the second pair of forceps is inserted between the 3rd and 4th sternite or tergite and the sclerites are gently pulled away going posteriorly down the gaster until the female reproductive system is accessible. Differentiating specific reproductive organs from each other in a winged female can be difficult because of their inconspicuous shape and position along with their clear and transparent tissues. However, it is easy to differentiate from the digestive system due to visible contents within the parts of the digestive system. The ovaries are the most conspicuous because of the distinctly white-ovoid developing ova. Once the ovaries have been located, gently follow the ovaries to the uterine pouch continuing until the bursa copulatrix is located. This may require removal of all sternites and tergites along with the digestive system, fat bodies, accessory tissues, venom sac, Dufour’s gland, and sting. If the droplet becomes cloudy because of the dissection process, gently pick up the intact reproductive system and place in a new droplet of water to continue the removal of the non-essential pieces. After the removal of all non-reproductive system organs, tissues and structures the intact reproductive system should remain. Gently grasped the reproductive system with one pair of forceps and lift the entire structure out of the droplet of water. Once removed, the reproductive system is placed in a clean drop of water to remove possible contaminants, then with a pair of forceps it is lifted out and placed on a dry portion of the glass slide. Touch the tip of a pair of forceps to the reproductive system to remove excess water via capillary action. Then place the female reproductive system into in an autosampler vial containing a 200 µL glass insert containing 30 µL of methanol (Merck, SupraSolv for gas Chromatography-Mass Spectroscopy, Billerica, MA). The tissue was crushed with forceps, and another 30 µL of methanol was used to rinse the forceps into the autosampler vial. This was followed by sonicated and vortex mixing. The reproductive tract extract was centrifuged (800 rpm, 2 min) and the supernatant was injected (1 µL) on an Agilent Intuvo 9000 GC system (Santa Clara, CA) equipped with an HP-5 423MS ultra-inert non-polar column, 30 m × 0.25 m i.d. column, coupled to a 5977 B GC-MS instrument and method “b”. The resulting total ion chromatograms were subjected to specific fragment ion analysis: m/z 97, 107, 120 (base peak), and 164. These fragments are specific and diagnostic for tyramides (low picograms can be detected). Before and after these samples were analyzed tyramides were detectable in other samples. This process was repeated for 10 unique dissections.

### Tyramides and the mating process

The following experiment was designed to test whether fire ant males release tyramides (from the endophallic bladder) simultaneously with the accessory gland and seminal vesicle contents or after accessory gland and seminal vesicle contents have been transferred (see Supplementary Fig. 2a). Newly mated fire ant queens were collected from a concrete pad (9.5 × 12.5 m) within two minutes of landing and dealation (wing removal). They were immediately placed in a chilled beaker in an insulated container with ice. Within 10 seconds the NMQs were immobile. Previously, a set of GC-MS auto sampler vials (2 mL) containing 150 μl inserts were prepared along with another set of auto sampler vials without inserts. Individual chilled NMQs were selected based on large gaster size (high weight is correlated with the monogyne fire ant social form^36^ picked up with feather forceps (BioQuip Products Inc., Rancho Dominguez, CA) by the thorax and held over an auto sampler vial with insert, such that the tip of the NMQ’s abdomen touched the inside of the insert. A Hamilton 100 μl syringe (Cole-Palmer, Vernon Hills, IL) was used to release 30 μl of methanol over the NMQ’s gaster tip and into the insert (see Supplementary Fig. 2b). The gaster tip washed NMQ was then placed in a chilled autosampler vial (without insert) for further analyses. The above procedure was replicated with six NMQs. The selected NMQs were brought back to the laboratory and weighed (Ohaus GA200D Balance, Parsippany, NJ, USA) within an hour of collection. One of the NMQs was low weight (<15 mg); therefore, these results were discarded. The NMQ abdominal tip methanol wash was analyzed and quantified by GC-MS (“b”) for tyramides using the External Standard (ES, butanoyl tyramide) method (Fig. 2a, *n* = 5).

### Tyramides in newly mated queens—collection

On June 4, 2021, conditions were good for a fire ant mating flight: rain (the day before), wind (<5 Km/h), and temperature (24–33 °C)^10^. The objective was to collect NMQs immediately upon landing with wings present, place them each in a 7 mL scintillation vial (DWK Life sciences, Wheaton, Millville, NJ), and age them at ambient outside temperature/humidity (24–29 °C, 70–90% humidity) for the following time periods: 0, 3, 6, 9, 12, 15, and 18 min (a probing experiment showed that tyramides were absent in NMQs 30 min after collection). One person was managing the replicates (12) for each time. Four assistants collected newly mated queens (with wings) from a parking lot in Gainesville, FL. Immediately upon placing a NMQ into a vial, a stopwatch was started and the NMQ was brought directly to the manager. The assistant obtained another vial and stopwatch for the next NMQ collection. For time zero the manager pushed the vials directly into a cooler with crushed ice to stop potential enzymatic activity. After time zero, the manager kept the newly collected NMQ and stopwatch to monitor sample time to fill the six post NMQ landing time periods. When each NMQ’s designated time ended, its vial was pushed into a cooler filled with crushed ice to halt enzymatic hydrolysis of tyramides. That stopwatch was then available for another NMQ collector. After all time periods had 12 replicates the 7 mL vials preserved in ice were placed in a refrigerator (4 °C). Within 24 h, all NMQs were weighed. A similar NMQ experiment provided an additional pool of timed NMQ samples. Only those NMQs that weighed ≥15.0 mg (Denver Instrument M-220D or Ohaus GA 200D, calibrated yearly) were moved forward to the chemical analysis part of the experiment. Mating flights in the Gainesville area are characterized by having a mixture of monogyne and polygyne-derived NMQs. The monogyne NMQs are defined as being ≥15.0 mg and polygyne derived NMQs can range from 9 to 14 mg. The number of replicates for each time was variable due to the NMQ weight criteria. Replicate numbers for the 7 time periods were: 11, 8, 6, 4, 10, 4, 4, respectively.

### Loss of tyramides in newly mated queens—chemical analyses

The NMQs ≥ 15.0 mg were removed from their vials and two aliquots of 30 µL of methanol were expressed over the gaster tip of the NMQ using a micro syringe (100 µL), such that the methanol went into the original 7 mL vial. This procedure captured tyramides on the NMQ gaster plus tyramides that the NMQ may have inadvertently put on the surface of the vial (see Supplementary Fig. 2b). The live NMQ was immediately put in a new 7 mL vial cooled in crushed ice. The vials with methanol rinsed NMQs were returned to a refrigerator. Within 24 h for each NMQ, the head and thorax were removed and discarded, and the gaster was quickly ruptured with forceps and placed into a GC-flat bottom autosampler vial. The forceps were rinsed once with methanol (50 ml) directly into the vial. The NMQ gaster was disintegrated with a Sonic Dismembrator (Fisher Scientific-model 100, Waltham, MA USA) (for 30 s). The probe was rinsed with methanol (3x50ml) into the vial. Then synthetic butanoyl tyramide^20^ (400 ng) was added as an internal standard and the contents of the vial sonicated for 15 s (Ultrasonicator, Branson, Model 3510, Qsonica, LLC, New Town, CT USA) and vortexed for 15 s (Fischer Scientific-Digital Vortex Mixer). The resulting methanol extract was transferred to a centrifuge tube (0.5 ml) and centrifuged (Eppendorf 5452 Minispin Centrifuge, Fisher Scientific, Dubuque, IA) for 2 min at 5000 rpm. The supernatant was carefully transferred via syringe to a new GC-autosampler vial for qualitative and quantitative analyses (Fig. 2b, *t* = 0, *n* = 11). This process was repeated until all qualified replicates were completed, see Fig. 2d for results. Replicate numbers for the 7 time periods were: 11, 8, 6, 4, 10, 4, 4, respectively.

### Tyramides in newly mated queen spermatheca

The spermatheca in an inseminated fire ant queen is a conspicuous white-yellow kidney bean-shaped structure located near the posterior end of the gaster. The dissection procedure: A newly mated queen was placed on a glass slide (with a few 40 μL droplets of DI water) and the gaster was separated from the rest of the body by pulling in opposite directions on the petiole with two pairs of forceps. During dissection, the gaster is stabilized via: (A) If the petiole is still attached to the gaster, it can be used to stabilize the gaster during dissection, or (B) if the petiole is not attached, the gaster can be grasped by the first sternite or tergite with a pair of forceps. For A or B, the second pair of forceps is inserted between the 3rd and 4th sternite or tergite and the sclerites are pulled away going posteriorly down the gaster until the female reproductive system is accessible. Unnecessary tissues and organs are removed until the spermatheca is located. Then enough tissues and remaining organs are separated and removed until the spermatheca can be isolated and removed by placing a pair of forceps under it and lifting upwards, while the other forceps is used to hold down the remaining contents. Once removed, the spermatheca is placed in a clean drop of water to remove possible contaminants, then the spermatheca is lifted out with a pair of forceps and placed on a dry portion of the glass slide. The tip of a pair of forceps was touched to the spermatheca to remove excess water via capillary action, then the spermatheca was placed into an GC autosampler vial containing a glass insert that contained 30 µL of methanol. Another 30 μL of methanol was used to rinse the forceps into the glass insert. Synthetic butanoyl tyramide^20^ (130 ng) was added as an internal standard and the contents of the insert were sonicated for 15 s (Ultrasonicator, Branson, Model 3510, Qsonica, LLC, New Town, CT USA) and vortexed for 15 s (Fischer Scientific-Digital Vortex Mixer). The resulting methanol extract was transferred to a centrifuge tube (0.5 ml) and centrifuged (Eppendorf 5452 Minispin Centrifuge, Fisher Scientific, Dubuque, IA) for 2 min at 5000 rpm. The supernatant was carefully transferred via syringe to a new GC-autosampler vial for qualitative and quantitative analyses. No tyramides were detected in NMQ dissected/extracted spermatheca (*n* = 5). Before and after these samples were analyzed, tyramides were detectable.

### Enzymatic hydrolysis of tyramides—vulva/bursa copulatrix

To test the hypothesis that loss of tyramides in the NMQ reproductive system is under enzymatic control we devised the following dissection method for isolating the winged female vulva/bursa copulatrix. A drop of RNA/DNA free water was placed on a microscope slide. The gaster of a winged female was placed in the water droplet and opened using two pair of forceps, exposing the female reproductive system, digestive tract, sting and venom gland and sac. The digestive tract was removed first, along with as much cuticle and fat bodies as possible. The ovaries were removed next along with other tissue not associated with the vulva/bursa copulatrix. The venom sac is removed last. The sting remained attached to the vulva/bursa copulatrix. This combination was transferred to a GC flat bottomed insert (350 μL) and RNA/DNA free water (50 μl was added The vulva/bursa copulatrix dissection was repeated with another winged female and the resulting vulva/bursa copulatrix was put into the vial with the first. In all enzyme degradation experiments, two vulva/bursa copulatrix were used per reaction. The tissue/water in the vial was sonicated (Ultrasonicator, Branson, Model 3510) for 15 s. Then 50 μl of an aqueous solution of either synthetic acetyl- or hexanoyl-tyramide (80 μg/ml: 4 μg) was added and the mixture incubated (Isotemp Model 2001 Dry Heat Block, Fisher Scientific, Inc., Dubuque, IA and/or Eppendorf Thermomixer R, Fisher Scientific, Inc., Dubuque, IA) for 60 min at the following temperatures: Acetyl-tyramide (18, 20, 21, 25, 29, 32, 35 38, 40, 42 °C) and Hexanoyl-tyramide (20, 22, 24, 26, 28, 30, 32, 34, 36, 38, 40, 42 °C). Not all incubation temperatures are shown in Fig. 3a, b to improve visual clarity. During the 60 min time aliquots (5 μl) were removed at 5 min intervals and added to methanol (195 μl) to quench potential enzymatic activity. The *t* = 0 and subsequent 5 min aliquots (12 data points) were analyzed qualitatively and quantitatively for acetyl- or hexanoyl-tyramide by GC-MS (Instrument #2). The peak area calculated for *t* = 0, acetyl- or hexanoyl-tyramide was used as the 100% value. As the 60 min of potential enzyme reaction time progressed the peak area decreased proportionately to its decreased concentration. These values are presented as percent remaining in Fig. 3a, b for acetyl- and hexanoyl tyramide, respectively. Variability of the experimental process are most noticeable when no hydrolysis takes place, e.g., 20 and 42 °C, resulting in variable values around 100%.

### Enzymatic hydrolysis of tyramides—winged female digestive tract

A winged female was pinned ventrally into a silicone base as in the male reproductive system dissection procedure. The gaster was separated from the rest of the winged female above the petiole and placed in a 40 ul droplet of nuclease-free water on a glass slide. Using forceps, the sternites and tergites of the gaster were peeled away from the gastral contents. Once the cuticle was completely removed, the intact digestive tract (crop, midgut, and hindgut) was located and separated from fat bodies and reproductive tract. Gentle manipulation of the forceps was necessary to separate fat bodies from the digestive tract without perforating any of the digestive system. As necessary the dissected material was picked up with forceps and placed in a new droplet of water to remove fat bodies, other separated tissues, and/or for better visibility. After complete separation of fat bodies and reproductive system, the digestive tract was removed and placed in another clean droplet of water and gently agitated as a final “rinse”. It was then removed from the droplet with a pair of forceps while a second pair of forceps was used to remove excess water via capillary action (see male reproductive system dissection procedure) and placed in a GC flat bottomed vial insert (350 μL). RNA/DNA free water (50 μl) was added in preparation for incubation with tyramides. The tissue/water in the vial was sonicated (Ultrasonicator, Branson, Model 3510) for 15 s. Then 50 µl of an aqueous solution of either synthetic acetyl- or hexanoyl-tyramide (80 µg/ml: 4 µg) was added and the mixture incubated (Isotemp Model 2001 Dry Heat Block, Fisher Scientific, Inc., Dubuque, IA and/or Eppendorf Thermomixer R, Fisher Scientific, Inc., Dubuque, IA) for 60 min at the following temperatures: Acetyl-tyramide (32 and 35 °C) and Hexanoyl-tyramide (32 and 36 °C). During the 60 min period aliquots (5 µl) were removed at 5 min intervals and added to methanol (195 µl) to quench potential enzymatic activity. Quantitation of the *t* = 0 and subsequent 5 min aliquots (12 data points) were analyzed qualitatively for acetyl- or hexanoyl-tyramide by GC-MS (Instrument #2). The peak area calculated for *t* = 0, acetyl- or hexanoyl-tyramide was used as the 100% value. The experiment was repeated 4 times (two tyramides and two temperatures). Forty-eight samples were analyzed for tyramide degradation. As the 60 min of potential enzyme reaction time progressed the peak area did not change from the value obtained at *t* = 0. Therefore, incubation of tyramides with an extract of winged female digestive systems did not result in tyramide degradation. See Supplementary Fig. 4a.

### Evaluation of tyramide stability when incubated with water

RNA/DNA free water (50 µl) was added to a GC flat bottomed insert (350 µL). Then 50 µl of a solution of either synthetic acetyl- or hexanoyl-tyramide (80 µg/ml: 4 µg) was added, sonicated (Ultrasonicator, Branson, Model 3510) for 15 s and the solution incubated (Isotemp Model 2001 Dry Heat Block, Fisher Scientific, Inc., Dubuque, IA and/or Eppendorf Thermomixer R, Fisher Scientific, Inc., Dubuque, IA) for 60 min at the following temperatures: Acetyl-tyramide (32 and 35 °C) and Hexanoyl-tyramide (32 and 36 °C). During this period aliquots (5 µl) were removed at 5 min intervals and added to methanol (195 µl) to quench potential tyramide decomposition. Quantitation of the *t* = 0 and subsequent 5 min aliquots (12 data points) were analyzed qualitatively for acetyl- or hexanoyl-tyramide by GC-MS (Instrument #2). The peak area calculated for *t* = 0, acetyl- or hexanoyl-tyramide was used as the 100% value. The experiment was repeated 4 times (two tyramides and two temperatures). Forty-eight samples were analyzed for tyramide degradation. As the 60 min of potential tyramide decomposition progressed the peak area did not change relative to the *t* = 0 peak area. Therefore, incubation of tyramides in water did not result in hydrolysis or other decomposition. See Supplementary Fig. 4b.

### Qualitative analysis for tyramine

An Advion™ compact mass spectrometer, with an electrospray ionization (ESI) source operated in positive ion mode, and *Touch Express*™ Open Port Sampling Interface, and expression CMS data handling software (Advion, Ithaca, NY USA), was used to determine if tyramine was a product associated with the loss of tyramides after they were incubated with extracts of the winged female/vulva/ bursa copulatrix. The electrospray solvent was 0.1% formic acid in methanol at a flow rate of 100 μl/min. Initially, a tyramine standard was sampled (15 μl of a 4 ng/μl solution) and its profile characterized via software. Then the post tyramide/winged female/vulva/ bursa copulatrix incubation product(s) was prepared assuming all the tyramides were converted to tyramine. Five μl of a 4 μg/50 μl solution was diluted with 95 μl of methanol to approximate a potential tyramine concentration of 4 ng/μl, if all the tyramide was converted to tyramine. The diluted sample (15 μl) was introduced into the mass spectrometer and the output compared with the tyramine control.

### Qualitative and quantitative analysis for tyramine

The data presented in Fig. 3c is from a global metabolomics study (Southeast Center for Integrated Metabolomics, University of Florida, Gainesville, FL) in part comparing winged females and newly mated queens (NMQs). Samples analyzed: Winged females >15 mg in weight were collected from monogyne field colonies (Gainesville, FL). Newly mated queens (NMQ) were collected directly after they landed from natural mating flights (Gainesville, FL area) and weighed. NMQs >15 mg were selected. Winged females and NMQ samples were kept in a freezer (−80 °C). Just prior to analysis winged females and NMQs were taken individually from freezer storage, then head, thorax, and gaster were dissected and each placed in a labeled centrifuge tube, and immediately returned to the freezer (−80 °C). The process was repeated until there were *n* = 7 NMQs and *n* = 6 winged females for the two categories. Samples (39 samples total) were transported to the Metabolomics Center on Dry-Ice. All samples were extracted following the Metabolomics Center’s cellular extraction procedure, pre-normalization to sample protein content of 100 μg/mL. Global metabolomics profiling was performed on a Thermo Q-Exactive Oribtrap mass spectrometer with Dionex UHPLC and autosampler. Each sample was spiked with 10 μl of 4 μg/mL 4-hydroxy-3-methoxy-benzylamine as an internal standard (IS) for relative quantitation of the biogenic amines, There were four replicates of winged females and NMQs for which results were obtained for both tyramine and octopamine. There were three additional replicates for tyramine—*n* = 7 for tyramine and *n* = 4 for octopamine. All *n* values represent biologically independent samples. The final concentration of the IS in each LC-MS sample was 800 ng/mL. All samples were analyzed in positive and negative heated electrospray ionization with a mass resolution of 35,000 at m/z 200 as separate injections. Separation was achieved on an ACE 18-pfp 100 × 2.1 mm, 2 µm column with mobile phase A as 0.1% formic acid in water and mobile phase B as acetonitrile. The flow rate was 350 μL/min with a column temperature of 25 °C. Four μL was injected for negative ions and 2 µL for positive ions. Relative quantitation of tyramine and octopamine was done in the positive mode. The relative internal standard method was used for quantitation.

### Winged female injection

Injections were carried out using a UMP3 Microsyringe Injector and Micro4 Controller with a Nanoliter 2010 for microprocessor controlled nanoliter injection (World Precision Instruments, Sarasota, FL). Winged females (>15 mg) were collected from monogyne field colonies (Gainesville, FL). Just prior to injection, the winged females were temporarily immobilized by chilling them for 1 min in a freezer. A pulled capillary needle was inserted into the 7th abdominal segment of a chilled winged female. A total of 100 nL of a 1% tyramine/water or saline solution was injected into the winged female in two ×50 nL aliquots (100 nL in one injection could lead to back pressure and inaccurate delivery of the treatment or control, delay in between aliquots was <10 s). Once each injection process was complete the winged female was observed for normal movement. Controls consisted of the same procedure as above except water or saline was injected. Winged females showing any sign of injury after injection were discarded, as were injected winged females that died within three days of injection. Each successfully injected winged female (treatment and control) was placed into their own nuptial chamber prepared from a 20 ml test-tube (15 cm long, 1.5 cm i.d., Fisher brand, Dubuque, IA) with water (6 cm), cotton ball (2 cm), Castone^®^ floor (2 cm), and a cotton ball (2 cm) at the open end to create a chamber (3 cm long × 1.5 cm i.d.).

### Time to wing loss

The winged female controls (injected with water, *n* = 14) and treatments (injected with tyramine in water, *n* = 7) were placed in nuptial chambers and were observed for wing loss every 24 h for seven days. Wing loss is defined as complete loss of wings (some alates would only lose one or parts of their wings). The data were visualized using Kaplan−Meier survival curves. See Fig. 4a. All *n*/*N* values represent biologically independent samples.

### Poison sac dissection

An experimental winged female (post injection) was taken for the dissection procedure described here within 12 h of meeting the criteria of both egg-laying and wing loss. The female sexual was held at the petiole using forceps (Dumont No.5, sharpened to fine points) and placed on a watch glass (filled with water) facing ventral side up. One pair of forceps were used to grasp the base of the sting apparatus and a second forceps grasped the last segment of the gaster and this segment was gently pulled away from the dealated winged female. The poison sac, Dufour’s gland, pair of connected ovaries, and fat bodies were exposed. After the fat bodies were removed, forceps were used to grasp and pinch off the narrow tip (posterior end) of the poison sac. The poison sac then was placed in a GC-autosampler vial insert (200 μL) containing 30 μl of hexane (Fisher Optiva, Cole Palmer, Vernon Hills, IL). The queen poison sac is the source of worker attractant pheromones^24^. More abundant polygyne queens were collected and their poison sacs dissected and prepared as described above for use as positive controls.

### Queen pheromone production bioassay

Worker ants (100) were placed in a container (VERSAtainer brand restaurant, 160 mm × 40 mm × 100 mm, Pactiv LLC, Lake Forest, IL USA), at least 30 min prior to the start of the experiment. The upper half of the inner sides of the container were painted with Fluon™ to prevent escape. Two phase separation filter paper squares (1 × 1 cm^2^) for treatment and control, (Whatman brand, Fisher Scientific, Inc., Waltham, MA USA) were placed on aluminum foil squares (1.5 × 1.5 cm) located at each end of the tray. The venom sac hexane extracts were from: (a) Saline injected winged females (WF, *n* = 8), (b) Tyramine injected winged females (WF+, *n* = 10), and (c) Polygyne fire ant queens (*n* = 12) (see poison sac dissection, above). All *n*/*N* values represent biologically independent samples. The concentrations were standardized so that 10 μl of extract contained 0.33 venom sac equivalents. The bioassay position of a venom sac extract was randomly decided. The control (10 μl of hexane) was placed on the remaining piece of filter paper. The observer immediately started a timer and recorded the number of ants on treatment and hexane control pieces of filter paper every 2 min for 10 min. The mean number of ants on the filter paper squares for the 5 counts were used for statistical analyses (see Fig. 4b).

### Ovariole dissection-area measurement

After poison sac removal (see above), the ovary pair was carefully separated, removed, and placed in a water droplet on a separate glass slide (75 × 25 × 1 mm), then the ovaries were covered with a pre-cleaned microscope glass cover slip (18 × 18 mm square, Fisher Scientific, Dubuque, IA) to flatten them to the surface of the slide. This provides a uniform method for measuring the two-dimensional area of the ovary surface. The surface area of each ovary pair (see ovariole and poison sac dissection) was measured by pixel count using a microscope (Model-Leica MDG32) attached to a digital microscope color camera (Leica DFC 295), connected to a computer running Leica Application Suite V4 with ImageJ v1.51 (Leica Camera, Inc., Allendale, N.J. USA). The Kilopixel area of treatments (*n* = 15) and controls (*n* = 15) were compared (see Fig. 4c).

### Reporting summary

Further information on research design is available in the Nature Research Reporting Summary linked to this article.

## Supplementary information

Supplementary Information

Description of Additional Supplementary Files

Supplementary Data 1

Reporting Summary

## Data Availability

Data generated or analyzed during this study are included in this published article and Supplementary Data 1.
