# Supplementary material for: Male fire ant neurotransmitter precursors trigger reproductive development in females after mating
Source: Commun Biol. 2021 Dec 15;4:1400. doi: 10.1038/s42003-021-02921-5 (PMC8674293; doi:10.1038/s42003-021-02921-5)
Supplement: Supplementary file 1 — Supplementary Information [file 42003_2021_2921_MOESM1_ESM.pdf]

COMMSBIO-20-3350B Vander Meer

Supplementary Figures

Male fire ant neurotransmitter precursors trigger reproductive development in females after mating

Robert K. Vander Meer, Satya P. Chinta, Tappey H. Jones, Erin E. O’Reilly, Rachelle M. M.

Adams

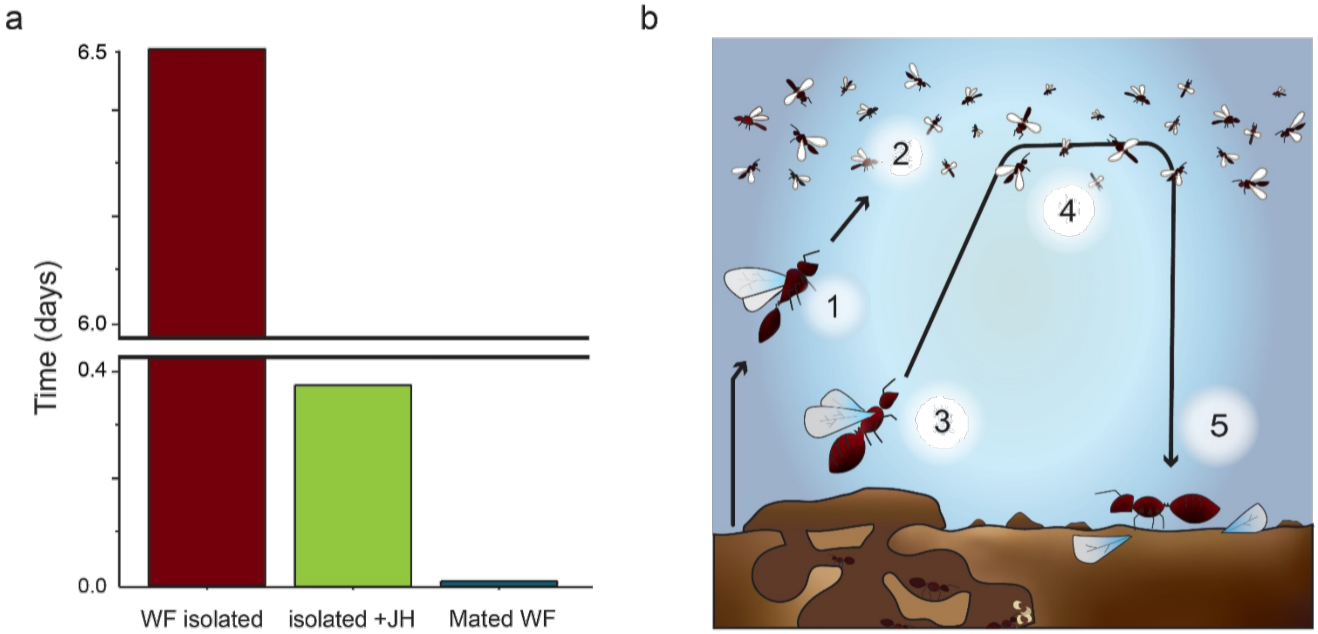

Supplementary Figure 1. **Reproductive development – the before and after mating dichotomy**. **a**: Comparison of the time-to-wing loss for winged females: 1. If removed from the influence of their queen (WF isolated), 2. The maximum effect of Juvenile Hormone III (JH) treatments on isolated winged females (isolated+JH). 3. Wing loss after mating via a mating flight (mated WF = newly mated queen). **b**: A diagram of events associated with mating flights. When weather conditions are right, excited males, winged females, and workers open the mound tumulus followed by flight activation: males fly first (1) and form a Lek (2), followed by winged female flights (3), and mating (4), then newly mated queens land and (5) attempt to start a new colony.

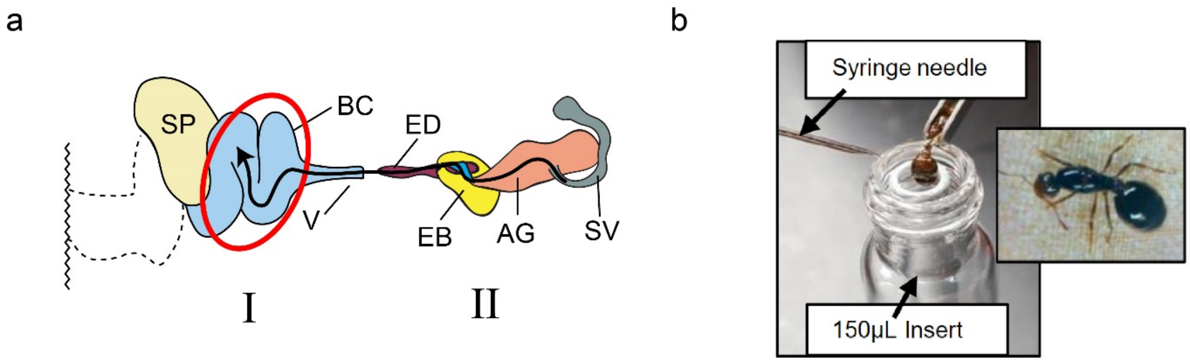

Supplementary Figure 2. **Release of tyramides. a-I**: Diagram of part of the winged female reproductive system, SP: spermatheca, BC: bursa copulatrix, V: vulva (see dissection of the female reproductive system in the Methods section). **a-II**: Diagram of the fire ant male reproductive system, ED: ejaculatory duct, EB: endophallic bladder, AG: accessory glands, and SV: seminal vesicles[22]. The reproductive systems are oriented as if in a potential mating configuration. The black arrow shows potential simultaneous material flow from male to female during mating. The red oval shows the area of the winged female reproductive system dissected for tyramide hydrolase experiments. **b**: The gaster tip of newly mated queens were rinsed with 30 μL of methanol with a syringe as depicted .in b, above. The collected methanol was analyzed for tyramides. Significant amounts of tyramides were detected on the outside of the gaster (see Fig. 2a), in support of tyramide release last rather than first and simultaneously with the AG and SV contents, as suggested by the black arrow in **aI and aII**.

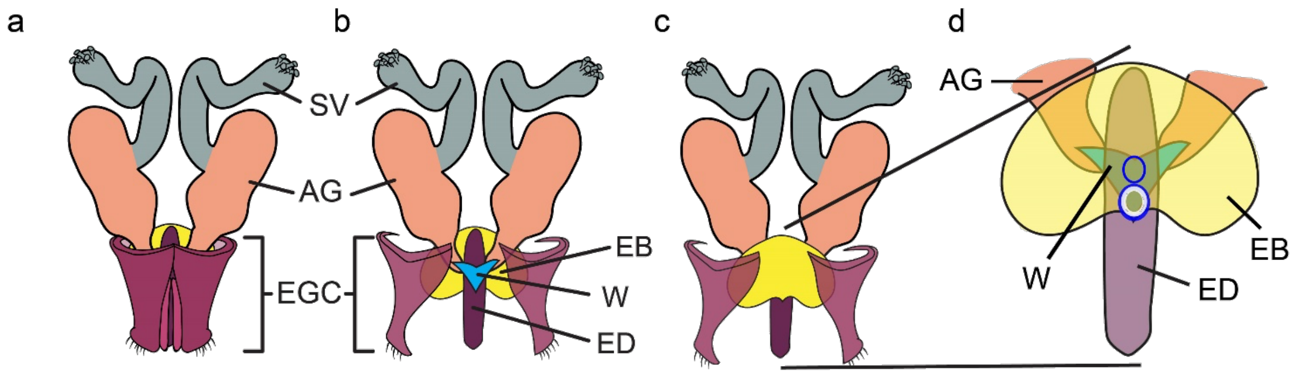

Supplementary Figure 3. **Male reproductive system - details.** **a**: Dorsal view of the male reproductive system showing external genitalia complex (EGC) and internal structures; seminal vesicles (SV) and accessory glands (AG). **b**: Dorsal view with laminae aedeagalis and laminae annularis removed and the laminae parameralis pulled laterally to expose the sclerotized wedge (W) sitting dorsally to the ejaculatory duct (ED) and the endophallic bladder (EB). **c**: Ventral view of the reproductive system with the laminae parameralis moved laterally, showing the endophallic bladder as the ventral-most structure. **d**: Enlarged ventral view of the EGC showing two regions (blue ellipses) of entry into the ejaculatory duct (ED); the upper being where the accessory glands meet the anterior region of the wedge and the lower being where the endophallic bladder contents enter at the posterior region of the wedge. Drawings based on reference[22].

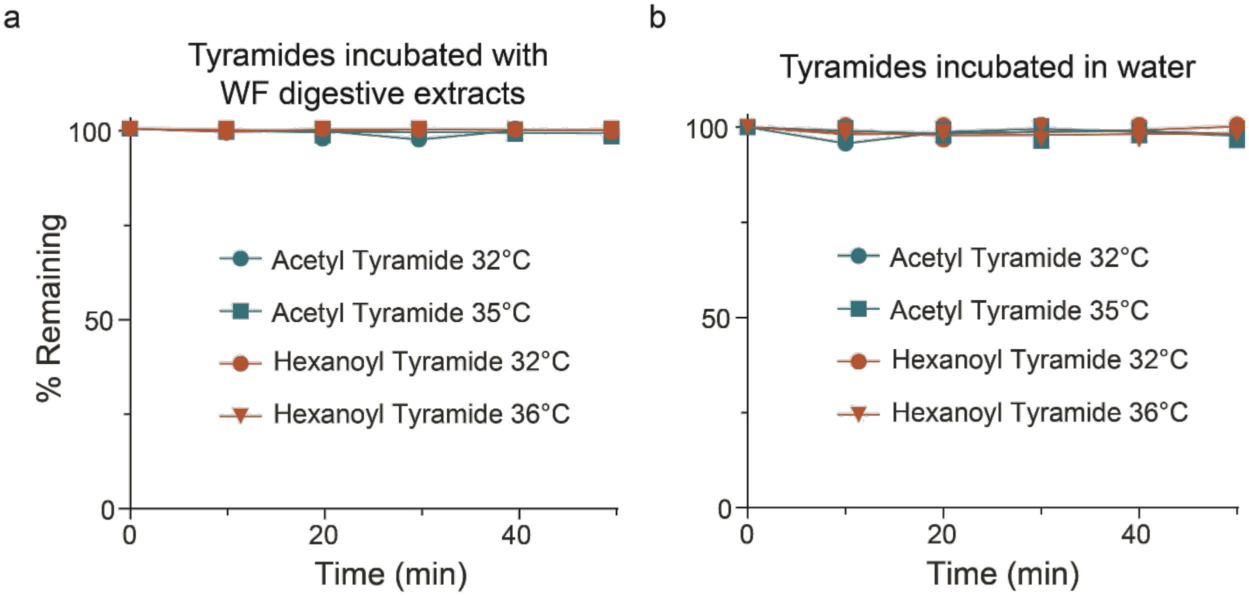

Supplementary Figure 4. **Tyramide hydrolysis - controls**: **a**: Incubation of tyramides in an aqueous extract of the digestive system from a winged female (WF) at temperatures that result in rapid hydrolysis when incubated with an aqueous extract of a winged female vulva/bursa copulatrix. **b**: Incubation of tyramides in water at temperatures that result in rapid hydrolysis when incubated with an aqueous extract of a winged female vulva/bursa copulatrix. No hydrolysis was observed when tyramides were incubated with (**a**) an extract of winged female digestive tract, or with **b**: water.
