# Supplementary material for: Male fire ant neurotransmitter precursors trigger reproductive development in females after mating
Source: Commun Biol. 2021 Dec 15;4:1400. doi: 10.1038/s42003-021-02921-5 (PMC8674293; doi:10.1038/s42003-021-02921-5)
Supplement: Supplementary file 2 — Description of Additional Supplementary Files [file 42003_2021_2921_MOESM2_ESM.pdf]

Description of Additional Supplementary Files

File name: Supplementary Data 1.
Description: This Excel file contains the data used to generate Figures 1, 2, 3, 4, and Table 1.
