# Supplementary material for: Male fire ant neurotransmitter precursors trigger reproductive development in females after mating
Source: Commun Biol. 2021 Dec 15;4:1400. doi: 10.1038/s42003-021-02921-5 (PMC8674293; doi:10.1038/s42003-021-02921-5)
Supplement: Supplementary file 4 — Reporting Summary [file 42003_2021_2921_MOESM4_ESM.pdf]

nature research

Corresponding author(s): Vander Meer COMMSBIO-20-3350A

Last updated by author(s): Nov 4, 2021

Reporting Summary

Nature Research wishes to improve the reproducibility of the work that we publish. This form provides structure for consistency and transparency in reporting. For further information on Nature Research policies, see our Editorial Policies and the Editorial Policy Checklist.

Statistics

For all statistical analyses, confirm that the following items are present in the figure legend, table legend, main text, or Methods section.

n/a	Confirmed	
☐	☒	The exact sample size (*n*) for each experimental group/condition, given as a discrete number and unit of measurement
☐	☒	A statement on whether measurements were taken from distinct samples or whether the same sample was measured repeatedly
☐	☒	The statistical test(s) used AND whether they are one- or two-sided<br>*Only common tests should be described solely by name; describe more complex techniques in the Methods section.*
☐	☒	A description of all covariates tested
☐	☒	A description of any assumptions or corrections, such as tests of normality and adjustment for multiple comparisons
☐	☒	A full description of the statistical parameters including central tendency (e.g. means) or other basic estimates (e.g. regression coefficient) AND variation (e.g. standard deviation) or associated estimates of uncertainty (e.g. confidence intervals)
☐	☒	For null hypothesis testing, the test statistic (e.g. *F*, *t*, *r*) with confidence intervals, effect sizes, degrees of freedom and *P* value noted<br>*Give P values as exact values whenever suitable.*
☒	☐	For Bayesian analysis, information on the choice of priors and Markov chain Monte Carlo settings
☒	☐	For hierarchical and complex designs, identification of the appropriate level for tests and full reporting of outcomes
☒	☐	Estimates of effect sizes (e.g. Cohen's *d*, Pearson's *r*), indicating how they were calculated

Our web collection on statistics for biologists contains articles on many of the points above.

Software and code

Policy information about availability of computer code

Data collection	No special software was used.
Data analysis	All statistical procedures and graphical representations were carried out using GraphPad Prism, version 9 (GraphPad Software Inc., San Diego, CA)

For manuscripts utilizing custom algorithms or software that are central to the research but not yet described in published literature, software must be made available to editors and reviewers. We strongly encourage code deposition in a community repository (e.g. GitHub). See the Nature Research guidelines for submitting code & software for further information.

Data

Policy information about availability of data

All manuscripts must include a data availability statement. This statement should provide the following information, where applicable:

- Accession codes, unique identifiers, or web links for publicly available datasets
- A list of figures that have associated raw data
- A description of any restrictions on data availability

All data associated with the Figures and Table are provided in Supplementary Data-1.

Field-specific reporting

Please select the one below that is the best fit for your research. If you are not sure, read the appropriate sections before making your selection.

☐ Life sciences ☐ Behavioural & social sciences ☒ Ecological, evolutionary & environmental sciences

For a reference copy of the document with all sections, see nature.com/documents/nr-reporting-summary-flat.pdf

Ecological, evolutionary & environmental sciences study design

All studies must disclose on these points even when the disclosure is negative.

Study description	The study revolves around a unique class of compound called Tyramides. Tyramides are readily identified and quantified via GC-MS techniques. Our interest in these compounds is that they are found only in males of a particular ant sub--family that includes economically important Solenopsis (fire ant) species. An almost 40 year-old problem revolves around fire ant queen inhibition of reproductive development in her sexual daughters via a primer pheromone. Sexual daughters removed from the queen's influence need up to 6 days to start reproductive development. In contrast, when those inhibited sexual daughters leave the colony on a mating flight and mate they almost immediately commence sexual development (<30min). Previous studies eliminated a number of possibilities. This study relates several unexpected discoveries revolving around tyramides that provide an explanation for the rapid onset of reproductive development in newly mated fire ant queens, e.g., 1) tyramides are only found males; 2) males release tyramides into winged females after sperm transfer; 3) winged females produce an enzyme that converts tyramides to the biogenic amine neuromodulator, tyramine; 4) the enzyme is active only at temperatures reported for mating flights; 5) tyramine levels are elevated in newly mated queens. Male and female sexual samples are readily available throughout the fire ant range. Population densities are up to 120 colonies/h. Each colony produces about 5,000 males/female sexuals/year. Mating flights can occur throughout the year, depending on weather conditions. Mating flights are population-wide events at an altitude of about 150 m, with millions of male and female sexuals. Newly mated females are readily collected directly after landing and in large numbers. Male and female sexuals can be readily collected prior to mating flights from their natal colonies (maturity is correlated with weight). Thus, obtaining independent samples was not difficult. The number of replicates was dictated by variance, and in the number of newly mated queens collected that met the monogyne newly mated queen weight criterium.
Research sample	Samples analyzed were male and female sexuals, and newly mated female sexuals from S. invicta (red imported fire ant) colonies. As stated in the previous section, the number of colonies available is very large and the sexuals produced can be found throughout the year, but most abundantly during Spring and Summer. The replicate is the colony. Within colony replicates would be considered pseudo replicates.
Sampling strategy	No sample size calculation was performed. Tyramide analyses were carried out on, e.g. female sexuals that weighed >15mg. Similarly, newly mated queens were used only if their weight was >15mg. Mature males were selected by weight also. These procedures, in turn, reduced variation in the chemical analyses. In behavioral bioassays – procedures were developed to minimize human and ant variability; however, variance of experimental results generally dictated the number of replicates needed.
Data collection	Support staff with or under the supervision of one or more of the authors conducted bioassays. Data was recorded directly into notebooks and/or Excel spreadsheets.
Timing and spatial scale	Mating flights and colony production of male and female sexuals primarily occurs in Spring and Summer. Colony workers are available year-round for behavioral bioassays.
Data exclusions	No data were excluded.
Reproducibility	All attempts to repeat experiments were successful. The methods are detailed and should be reproducible by someone experienced with social insects, especially ants. The very important dissections are very detailed, and the GC-MS methods are well detailed and reproducible.
Randomization	Male and female sexuals in a fire ant colony can be in various stages of development. We used weight as a measure of maturity (>15mg + mature) this Weight group is ready to fly when weather conditions are right and, therefore, make the closest comparison to newly mated queens. The time period between a female sexual's flight from the mother colony and mating (newly mated queen) is <30 minutes. NMQs are grouped into the same weight class: >15mg as the in colony winged females. Mating flights are population-wide events about 150 m in the air. The numbers of NMQs during a flight are huge - millions - after mating it would be extremely rare for two NMQs from the same colony to land in the same place; therefore, collected NMQs are likely unrelated.
Blinding	Blinding was not used in these experiments. Quantitation of tyramides and tyramine was accomplished through standard GC-MS software. The three behavioral bioassays: a) time to wing loss – there is nothing subjective here – the alates must lose all 4 wings; b) worker response to poison sac extracts – the ants responded decisively; c) Results for saline versus tyramine injects were unambiguous. In other types of bioassays, we have used blinding.

Did the study involve field work? ☒ Yes ☐ No

Field work, collection and transport

Field conditions	Fire ant colonies inhabit most disturbed habitats, including roadsides. This was the standard collection locations – roadsides in the

Field conditions	Gainesville, FL area. The best conditions are high humidity, recent rain, sunny, and temperatures between 22 and 40C. Roadside fire ant collection safety precautions are established.
Location	There are a multitude of collection sites in the Gainesville area. We could provide the Lat/Long coordinates for a representative number if useful.
Access & import/export	Collections on private property is always preceded by obtaining permission from owners. There is no import/export of fire ants for this study.
Disturbance	If a colony is removed the resulting hole is filled with dirt.

Reporting for specific materials, systems and methods

We require information from authors about some types of materials, experimental systems and methods used in many studies. Here, indicate whether each material, system or method listed is relevant to your study. If you are not sure if a list item applies to your research, read the appropriate section before selecting a response.

Materials & experimental systems

n/a	Involved in the study
☒	☐ Antibodies
☒	☐ Eukaryotic cell lines
☒	☐ Palaeontology and archaeology
☐	☒ Animals and other organisms
☒	☐ Human research participants
☒	☐ Clinical data
☒	☐ Dual use research of concern

Methods

n/a	Involved in the study
☒	☐ ChIP-seq
☒	☐ Flow cytometry
☒	☐ MRI-based neuroimaging

Animals and other organisms

Policy information about studies involving animals; ARRIVE guidelines recommended for reporting animal research

Laboratory animals	The animals we work with are insects-ants. There are no standards in place for insects; however, our ant colonies are well cared for using established protocols.
Wild animals	We do get ant colonies from the field or start colonies from field collected newly mated queens.
Field-collected samples	Colonies are maintained using long established protocols.
Ethics oversight	Approval is not required for insects at this time.

Note that full information on the approval of the study protocol must also be provided in the manuscript.
